# Identification of modifiable gait parameters predictive of simulated tibial stress-fracture risk in men and women

**DOI:** 10.3389/fphys.2026.1905928

**Published:** 2026-08-05

**Authors:** Sridevi Nagaraja, Jose E. Rubio, Manivannan Subramaniyan, Junfei Tong, Michael Baggaley, W. Brent Edwards, Jaques Reifman

**Affiliations:** 1 Department of Defense Biotechnology High Performance Computing Software Applications Institute, Defense Health Agency Research & Development, Medical Research and Development Command, Fort Detrick, MD, United States; 2 The Henry M. Jackson Foundation for the Advancement of Military Medicine, Inc., Bethesda, MD, United States; 3 Human Performance Laboratory, Faculty of Kinesiology, University of Calgary, AB, Canada; 4 The McCaig Institute for Bone and Joint Health, Cumming School of Medicine, University of Calgary, AB, Canada

**Keywords:** gait parameters, individualized models, musculoskeletal injury, sex-specific models, tibial stress fracture

## Abstract

**Objective:**

This exploratory study investigated the ability of biomechanical gait parameters to predict and stratify simulated tibial stress-fracture (SF) risk in young, healthy adults.

**Methods:**

We analyzed 30 modifiable gait parameters (both kinematic and kinetic) for 21 men and 20 women when they ran with external loads (0, 11.3, or 22.7 kg) and the associated tibial SF risks during a simulated 10-week basic combat training in the U.S. military. To identify informative parameters and predict SF risk of the tibia, we separately developed personalized linear mixed-effects models for men and women. To stratify the SF risk, we developed logistic regression (LR) models.

**Results:**

Out of all the 30 parameters, ankle joint reaction force was the most predictive for both men and women, followed by hip external rotation angle for men and hip adduction moment for women. Using these parameters, LR models yielded area under the curve values of 0.81-0.95. For kinematic parameters alone, ankle dorsiflexion angle was the most predictive for both men and women, followed by hip external rotation angle for men and hip flexion angle for women. The associated LR models yielded reasonable performance for women (0.72-0.82) but weaker performance for men (0.53-0.71).

**Conclusion:**

We identified modifiable gait parameters predictive of simulated tibial SF risk in men and women and developed models based on kinematic and kinetic parameters that adequately predicted at-risk individuals. In the future, upon independent validation, this work has the potential to help identify recruits who may benefit from individualized training regimens and proactive interventions to reduce injury risk.

## 1 Introduction

Musculoskeletal injuries continue to be the leading cause of medical impediment to the readiness of U.S. military forces (Knapik et al., 2012; Grimm et al., 2019; Molloy et al., 2020; Gun et al., 2021). These include stress fracture (SF) to the tibia, one of the most common overuse injuries in the military (Kardouni et al., 2021), whose risk is associated with both non-modifiable (e.g., height, sex, and bone geometry) and modifiable (e.g., physical fitness, load carriage, and gait pattern) factors (Pohl et al., 2008; Nindl et al., 2016; Gun et al., 2021; Milner et al., 2023; Foulis et al., 2025). While non-modifiable risk factors can help identify susceptible populations, for example, the incidence rate of SF to the tibia is two to four times higher in female Service members than in their male counterparts (Molloy et al., 2020), modifiable factors offer the opportunity to identify specific at-risk individuals and adjust their gait pattern and physical activity to help reduce injury risk (Meardon and Derrick, 2014; Yong et al., 2018; Sheerin et al., 2020). For instance, reducing the stride length while running with or without load carriage decreases the load to the bones and can potentially reduce tibial SF risk for both men and women, although the extent of the reduction is individual specific (Willy et al., 2016; Sundaramurthy et al., 2023). In fact, previous retrospective studies show that in female runners with a history of medically confirmed tibial SF injury, certain modifiable gait parameters, including peak rearfoot ankle eversion and peak hip adduction angles, are significantly elevated compared to healthy individuals (Milner et al., 2010). However, the linkage between these modifiable risk factors and SF injury is laden with considerable between-sex and inter-individual variability (Rubio et al., 2023; Sundaramurthy et al., 2023). While advances in wearable sensors show promise for real-time gait assessment and tibial loading estimation, we still lack validated tools to monitor and predict at the individual level how changes in gait patterns during training or field exercises affect tibial SF risk (Matijevich et al., 2020; Prasanth et al., 2021). We hypothesize that certain gait parameters are predictive of tibial SF risk in men and women running with or without load carriage and can potentially be used to identify at-risk individuals.

Our U.S. Department of Defense team previously developed an interdisciplinary (experimental and computational) approach to predict SF risk to the tibia at the individual level when young, healthy men and women ran with or without load carriage (Unnikrishnan et al., 2021; Rubio et al., 2023; Sundaramurthy et al., 2023; Tong et al., 2023). Using individual-specific measurements from 21 men and 20 women, we developed personalized musculoskeletal models to predict changes in biomechanical gait parameters, finite-element models to predict stress and strain in the tibia, and probabilistic models to predict tibial SF risk of each individual at the end of a 10-week basic combat training (BCT) (Department of the Army, 2020). We assessed the effect of stature and load carriage in women (Unnikrishnan et al., 2021), stride length in women of different statures (Sundaramurthy et al., 2023), stature and load carriage in men (Tong et al., 2023), and differences in running biomechanics between men and women carrying external loads (Rubio et al., 2023). We found that: women of different statures adjust their gait patterns differently when running with external load (Unnikrishnan et al., 2021); a 10% reduction in running stride length decreases tibial SF risk in women regardless of stature (Sundaramurthy et al., 2023); load carriage but not stature significantly affects the gait patterns in men (Tong et al., 2023); and as load carriage increases from 0.0 kg to 22.7 kg, women have a greater increase in SF risk than men (250% vs. 133%) (Rubio et al., 2023). Overall, epidemiological and experimental studies corroborate these findings. However, these results cannot be attained in the field for real-time predictions because the individual-specific measurements need to be collected in a laboratory setting and the computational requirements may exceed those of current edge-computing capabilities.

In this exploratory study, we aim to develop statistical, data-driven models that could potentially be used in the field to make real-time predictions of an individual’s SF risk to the tibia and determine whether the risk is low or high. To this end, we used the results from our previous studies (Unnikrishnan et al., 2021; Rubio et al., 2023; Tong et al., 2023) to identify modifiable biomechanical (kinematic and kinetic) gait parameters that could be used as predictors in personalized linear mixed-effects (LME) models to estimate tibial SF risk and as predictors in binary logistic regression (LR) models to classify an individual’s risk level as low or high. We developed models for the 21 men and 20 women separately because of the known sex-specific gait adjustments and between-sex variability when individuals run with load carriage (Rubio et al., 2023), while considering the results of our previously developed probabilistic model as the “reference” estimate of SF risk for comparison purposes. We performed the entire analysis twice: once using kinematic and kinetic parameters and a second time using only kinematic parameters. This second analysis allowed us to determine whether joint angles alone, which could be potentially measured in the field using inertial measurement units (IMUs) (Seel et al., 2014), would be sufficient to reasonably predict SF risk. This study offers a new approach to identify modifiable gait parameters predictive of SF risk as well as the capability to potentially identify susceptible individuals and intervene to help reduce injury risk, which can be evaluated in future efforts.

## 2 Materials and methods

### 2.1 Experimental and computational data generation

To develop data-driven models for men and women that predict an individual’s SF risk of the tibia based on modifiable gait parameters, we used as predictors kinematic and kinetic gait parameters previously computed based on measurements collected during two experimental studies performed at the University of Calgary (Unnikrishnan et al., 2021; Rubio et al., 2023; Tong et al., 2023). The study protocol was approved by the University of Calgary Conjoint Health Research Ethics Board (REB16-1475) on 9 Sep 2016 and by the Human Research Protection Office at the U.S. Army Medical Research and Development Command (A-17952.2a) on 27 Dec 2016. We recruited participants via convenience sampling through advertisements placed on the Faculty of Kinesiology, University of Calgary social media sites and on study flyers across various sites at the University of Calgary. As per our inclusion criteria, we required participants to be between 18 and 25 years of age, as demographically representative of military recruits (Gordon et al., 2014), self-reported experienced treadmill runners (exercising at least three times per week), and free from injuries limiting physical activity for at least three months before enrollment. We obtained a written informed consent from each participant before enrollment. Briefly, these studies involved 41 young, healthy adults (21 men and 20 women) (**Table 1**). For each participant, we collected a quantitative computed tomography (CT) image of their left tibia, after which they completed three running trials in a simple randomized order at a constant speed of 3.0 m/s on a level instrumented treadmill (Bertec Corporation, Columbus, OH), including trials with no load, an 11.3-kg (25.0-lb) load, or a 22.7-kg (50.0-lb) load. For the trials with load carriage, participants wore a vest strapped to their upper body with the load symmetrically distributed between the back and front, as representative of military operations (Knapik et al., 2012). During the trials, we collected motion-capture data using the full-body Plug-in-Gait model consisting of 42 retroreflective markers secured bilaterally on the participant’s body, including anatomical landmark and segmental-tracking markers on the arm, trunk, pelvis, thigh, shank, and foot, and synchronously collected force-platform data at 1,000 Hz for 20 seconds.

**Table 1:** Anthropometric measurements of 21 men and 20 women participants.

Sex	Age (years)	Mass (kg)	Height (m)	Foot length (m)	Body fat (%)	BMI (kg/m^2^)
Men	19.6 (1.1)	72.0 (6.2)	1.77 (0.06)	0.27 (0.01)	8.7 (1.9)	23.0 (1.9)
Range	18-21	60.0-83.7	1.62-1.87	0.25-0.29	6.0-13.2	19.3-26.3
Women	19.6 (1.0)	60.3 (6.2)	1.65 (0.08)	0.24 (0.01)	18.3 (3.0)	22.2 (1.8)
Range	18-21	47.7-71.8	1.49-1.76	0.22-0.26	14.5-26.4	19.0-25.3

The data are presented as means (1 standard deviation) or range. BMI, body mass index.

**Figure 1:**
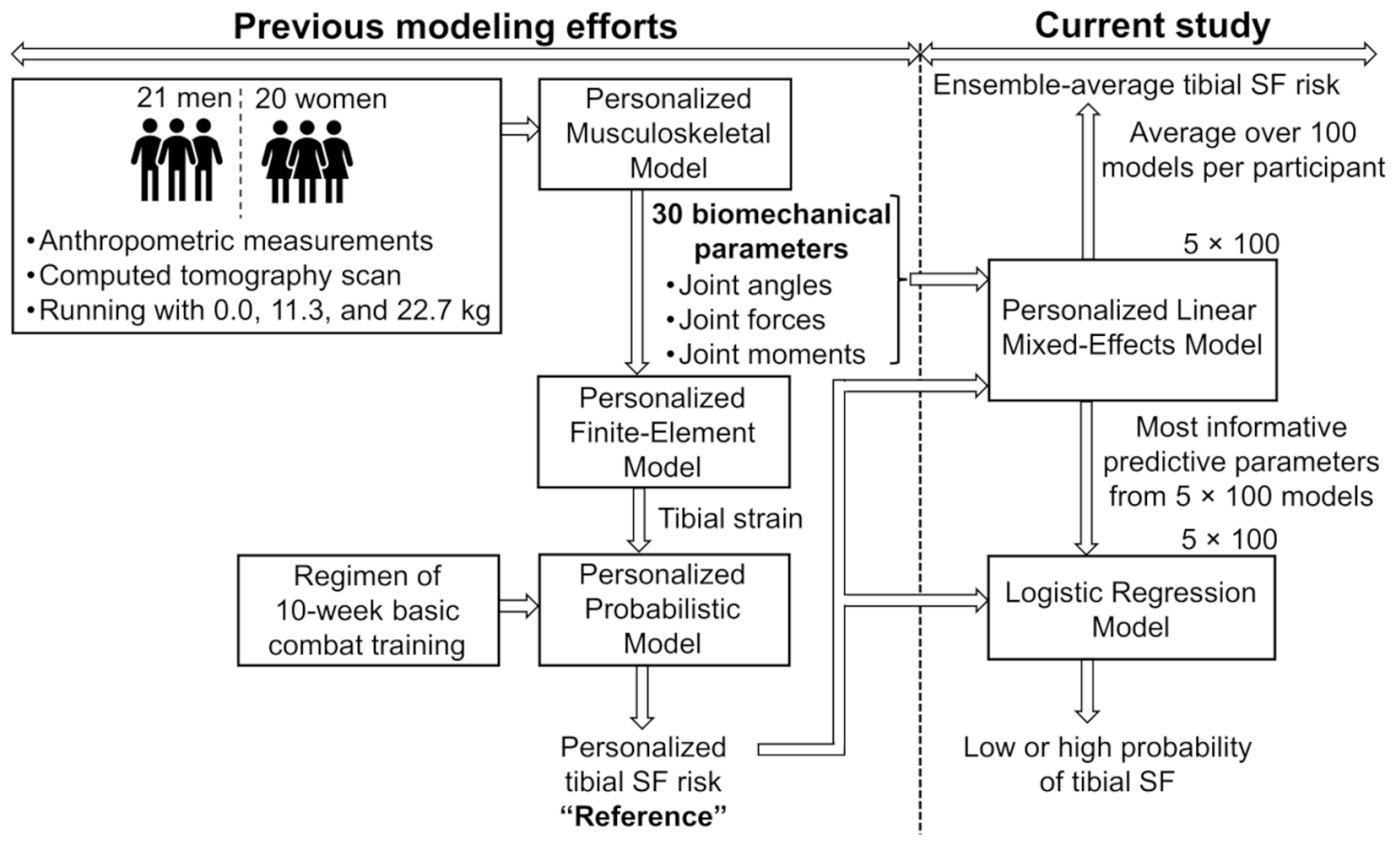
Previous modeling efforts and current study. In three previous efforts (Unnikrishnan et al., 2021; Rubio et al., 2023; Tong et al., 2023), we used participant-specific data from 21 men and 20 women running with load carriage (0.0, 11.3, or 22.7 kg) to develop personalized musculoskeletal, finite-element, and probabilistic models. These models estimated the values of 30 biomechanical parameters and predicted the tibial stress-fracture (SF) risk for each participant resulting from a simulated 10-week U.S. military basic combat training. In this study, we developed two data-driven statistical models. Considering the personalized probabilistic model predictions as a “reference” for determining SF risk, through a five-fold cross-validation procedure, we developed 100 linear mixed-effects (LME) models for each participant to identify the most informative biomechanical parameters and predict their ensemble-average SF risk. Then, using the top two most informative predictive parameters out of 500 LME models (5 folds × 100 models), we developed binary logistic regression models to predict whether a participant’s risk for tibial SF was low or high.

Using the CT image, anthropometric measurements, and experimental data, we previously developed personalized musculoskeletal models to compute joint kinematic (angles) and joint kinetic (moments and forces) parameters for each participant, under each of the three load-carriage conditions (Unnikrishnan et al., 2021; Rubio et al., 2023; Tong et al., 2023). Then, using the computed muscle forces, joint forces, and joint moments of each participant, we determined the input loading conditions and developed personalized finite-element models to predict the stresses and strains acting on the tibial bone, under each of the three loading conditions. Finally, using a probabilistic model that accounted for bone repair, adaptation, and fatigue (Edwards et al., 2010) and took the strains acting on the tibia as input, we predicted tibial SF risk for each participant under each condition at the end of a simulated 10-week BCT regimen (Department of the Army, 2020). **Figure 1** illustrates the steps used in our previous efforts to predict tibial SF risk. For additional information on the experimental protocols, regulatory approvals, and computational analyses, we refer the reader to the original articles (Unnikrishnan et al., 2021; Rubio et al., 2023; Tong et al., 2023).

Here, we assumed that the predictions of the probabilistic model in our previous efforts can serve as a “reference” SF risk against which to compare the data-driven models developed in this study. Previously, we observed good agreement between the model-predicted reference tibial SF risk after a simulated 10-week BCT and clinical observations, suggesting that personalized computational models that incorporated individual-specific anthropometric measurements, tibial geometry and material properties estimated through CT images, gait-parameter patterns acquired via motion caption, and ground reaction force measured by an instrumented treadmill, can adequately estimate tibial SF risk. For example, the model-predicted average risk for the three load-carriage conditions ranged from 4.5% to 15.7% in women and from 3.3% to 7.7% in men (Rubio et al., 2023), which is consistent with prior observations that the risk of SF in women is two to four times higher than in men (Knapik et al., 2012; Kucera et al., 2016; Molloy et al., 2020).

### 2.2 Modeling approach

In this study, we developed two data-driven statistical modeling approaches (**Figure 1**, right side). First, we developed personalized LME models to predict a participant’s tibial SF risk, and then we developed binary LR models to predict whether a participant’s risk was low or high. We used MATLAB R2022a (MathWorks, Natick, MA) to develop all the models.

#### 2.2.1 LME models to predict personalized tibial SF risk

**Figure 2:**
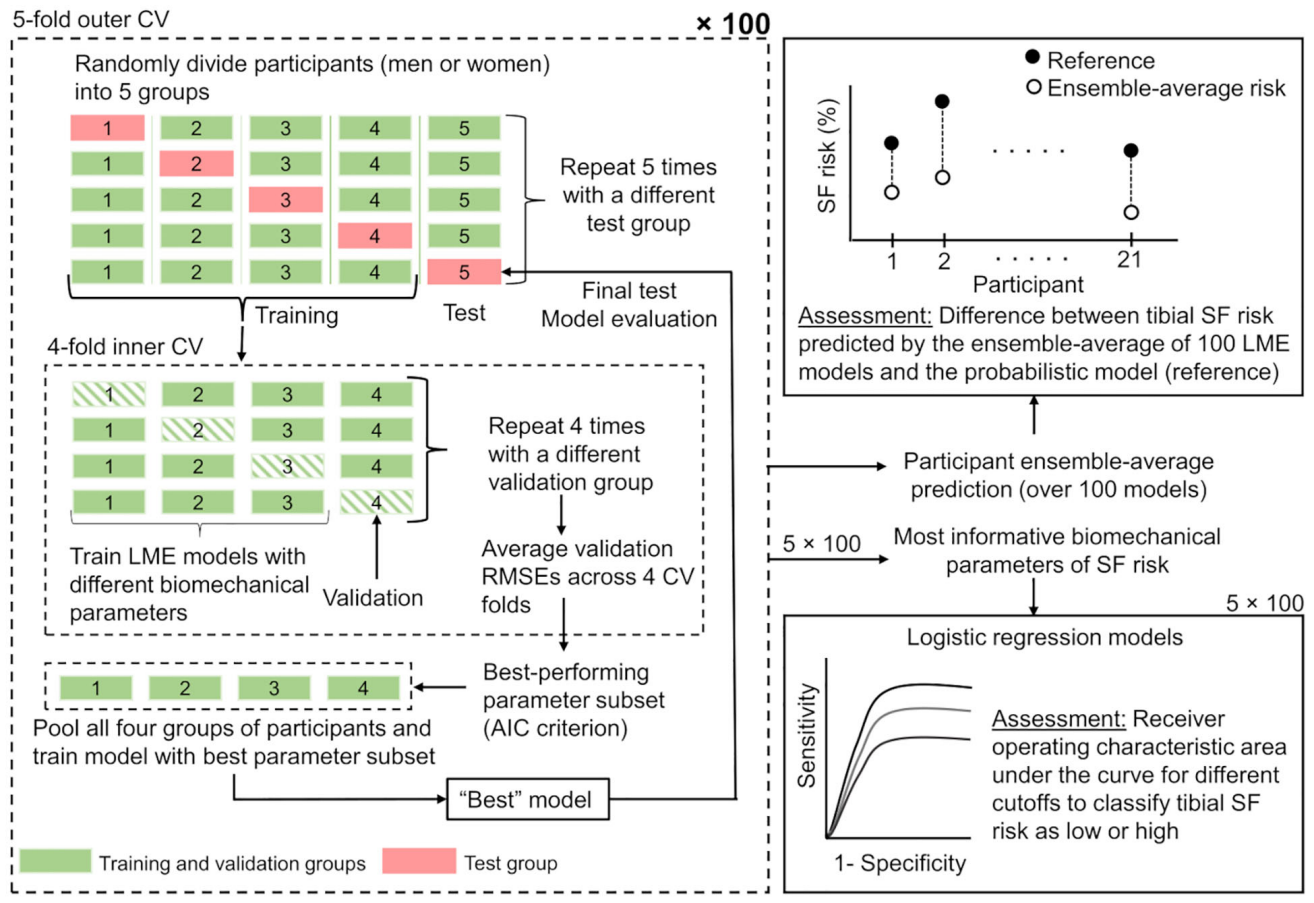
Five-fold nested cross-validation (CV) procedure for model selection (training and validation) in the “inner CV” and an “outer CV” for model evaluation (testing). We randomly divided participants (21 men and 20 women) into five groups of 4 or 5 participants per group and developed separate models for the men and women. For model selection in the inner CV, we trained linear mixed-effects (LME) models with different parameter subsets and selected the model with the best validation performance across the four CV folds to fit a new model (the “best” model) to the pool of all groups of participants in the inner CV. For model evaluation, we tested each of the five best models against the fifth group of each outer-CV fold. We repeated the entire procedure 100 times to construct 100 distinct models for each participant, for a total of 500 LME models (5 folds × 100 models). To predict the tibial stress-fracture (SF) risk for a participant, we averaged the 100 model predictions to generate an ensemble-average prediction, which we assessed by comparing against a reference SF risk value obtained by a previously validated probabilistic model (Unnikrishnan et al., 2021; Rubio et al., 2023; Tong et al., 2023). Finally, for men and women, we separately selected the most informative parameters to develop binary logistic regression models that classified participants as low or high risk for SF of the tibia. AIC, Akaike information criterion; RMSE, root mean square error.

We separately developed LME models for men and women to predict tibial SF risk for each participant, where we considered the peak values of 30 modifiable gait parameters (14 kinematic and 16 kinetic parameters) computed in our previous efforts discussed above as potential model predictors (**Table 2**). We did not normalize the gait parameter values, as LME models account for a varying range of predictor magnitudes by scaling the model coefficient accordingly (Harrison et al., 2018). In these models, we included a single random effect to account for the use of multiple data points (i.e., three, one for each loading condition) for each participant. Because tibial SF injuries are more prevalent in women than in men and gait biomechanics differ between sexes (Knapik et al., 2012; Rubio et al., 2023), we developed a separate set of models for men and women. We developed these models using a five-fold nested cross-validation (CV) procedure (**Figure 2**), consisting of an “inner CV” for model and parameter selection (training and validation) and an “outer CV” for model evaluation (testing). For model training, validation, and testing, we randomly partitioned the 20 women into five groups of 4 participants per group, where each group contained 12 data points (three sets of data from each of 4 participants). Similarly, we partitioned the 21 men into five groups of 4 or 5 participants per group. We performed all partitions at the participant level; the three data points contributed by each participant belonged to the group to which the participant was assigned.

**Table 2:** Biomechanical parameter number and description.

Parameter type	Parameter #	Description
Kinematic	1	Ankle dorsiflexion angle
	2	Ankle plantarflexion angle
	3	Subtalar eversion angle
	4	Subtalar inversion angle
	5	Trunk flexion angle
	6	Knee extension angle
	7	Knee flexion angle
	8	Knee stance flexion angle
	9	Hip abduction angle
	10	Hip adduction angle
	11	Hip extension angle
	12	Hip external rotation angle
	13	Hip flexion angle
	14	Hip internal rotation angle
Kinetic	15	Ground reaction force
	16	Ankle joint reaction force
	17	Ankle dorsiflexion moment
	18	Ankle plantarflexion moment
	19	Subtalar inversion moment
	20	Subtalar eversion moment
	21	Knee joint reaction force
	22	Knee extension moment
	23	Knee flexion moment
	24	Hip joint reaction force
	25	Hip abduction moment
	26	Hip adduction moment
	27	Hip extension moment
	28	Hip external rotation moment
	29	Hip flexion moment
	30	Hip internal rotation moment

To identify the most informative predictors (i.e., the most informative subset of model parameters), for each of the five outer-CV folds (**Figure 2**), we performed a four-fold inner CV (three groups for model training and one group for model validation), where we trained the models using different subsets of participants and gait parameters, as previously described (Subramaniyan et al., 2024). To reduce the potential for overfitting during model training and validation, where we used at least 48 data points (16 participants × 3 conditions), we limited the LME models to no more than five predictors. Briefly, in this iterative process, we developed models with one to five predictors using three groups of participants, computed the root mean square error (RMSE) between the model-predicted SF risk and the reference SF risk using the validation group, and averaged the RMSEs over the four inner-CV folds. We first assessed the models with a single parameter and progressively added the next most informative parameter, retaining only those subsets that improved model performance. At the end of the inner CV, we used the Akaike information criterion (AIC) (Cavanaugh, 2019), which balances the RMSE against the number of predictors, to identify the best-performing parameter subset (i.e., the model with lowest AIC value). Then, using this subset of parameters, we fitted a new model to all participants of the four groups pooled together and assessed the performance of this “best” model against the test (fifth) group of the outer-CV fold, which was not used for model selection in the inner-CV step. Accordingly, by repeating these steps five times, at the end of the five-fold CV procedure we obtained five “best” models.

To reduce the dependence of model selection and evaluation on a single random distribution of the participants, we repeated the five-fold CV procedure 100 times, using a different distribution of participants each time, resulting in 500 (5 × 100) different best LME models, each with its own parameter subset. Each time we repeated the five-fold CV procedure, each participant appeared in only one of the five test groups. Consequently, after 100 repetitions, we obtained 100 distinct models for each participant. Finally, to obtain the tibial SF-risk prediction for a specific participant, we created an ensemble consisting of all 100 models and averaged their predictions. This “ensemble-average” prediction, obtained only from models in which the information of the participant we wish to predict was held out during model selection, served as the predicted SF-risk value for that participant.

To identify the most informative parameters out of those selected to define the 500 best LME models, we counted the number of times each parameter appeared in the parameter subsets of the 500 models and computed their frequency. We designated the parameters with the highest and second highest frequency as the “most informative” parameters.

We repeated this entire procedure twice: once considering all 30 kinematic and kinetic parameters as potential LME model predictors and again using only the 14 kinematic parameters. The latter allowed us to investigate whether kinematic parameters alone were sufficient to provide accurate SF predictions.

#### 2.2.2 LR models to classify participants as low or high risk

To predict the probability of an individual being at “low” or “high” risk for a SF injury of the tibia, we separately developed binary LR models for men and for women (**Figures 1**, **2**). Because there are no established SF-risk thresholds for classifying risk as low or high, in the development of the LR models we investigated arbitrary cutoffs ranging from 2% to 8%, at 2% increments (**Supplementary Table S1**), to label the reference risk at the data level. As such, we labeled each of the three SF-risk values from each participant as “high” or “low” based on the different cutoffs. Above the 8% cutoff, the distribution of data points below (low risk) and above (high risk) this value became too unbalanced to warrant further analysis. We developed the LR models using the two most informative parameters identified by the fully nested CV procedure of the LME models described above, considering all 30 kinematic and kinetic parameters as well as only the 14 kinematic parameters. For each cutoff value, we performed a five-fold CV procedure 100 times utilizing the same distributions of participants as those generated for the LME models, to obtain 100 probability predictions from 100 LR models for each participant’s data point. We then computed the average prediction over the 100 results for each data point and used these average values to obtain the receiver operating characteristic area under the curve (AUC) for each cutoff. As with the LME models, through the CV procedure, we obtained the AUCs based on independent data not used in LR model development.

### 2.3 Statistical analysis

To independently assess the performance of the LME models for each man and each woman, we computed the absolute difference between the ensemble-average model-predicted SF-risk values and the corresponding reference values obtained with the probabilistic model. We then averaged the absolute differences over the three load-carriage conditions to generate one Mean Absolute Error (MAE) value per participant. As discussed above, we assessed the ability of the LR models to stratify low- and high-risk data points by computing the AUC and associated 95% confidence interval (CI) for cutoffs ranging from 2% to 8%, at 2% increments. As such, we designated data points below a cutoff as low risk and those above as high risk. We computed the CIs using the Wald method (Hanley and McNeil, 1982) assuming a symmetric normal distribution of the AUCs. Under certain conditions, the distribution can become skewed as the AUC approaches 1.00, leading to CIs exceeding 1.00. In such cases, we capped the upper bound of the CIs at 1.00.

## 3 Results

### 3.1 Most informative kinematic and kinetic predictors of SF risk in men and women

**Figure 3:**
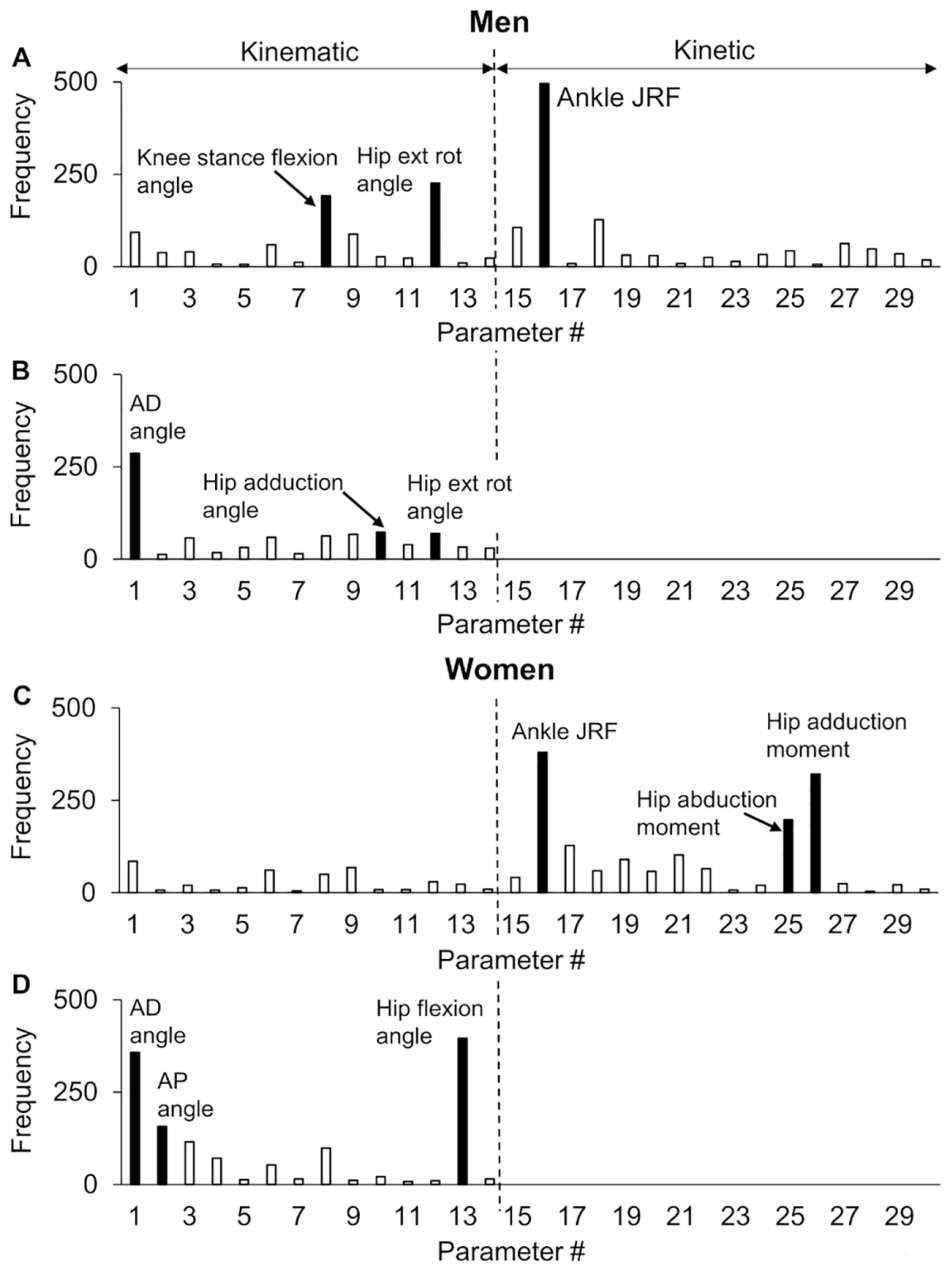
Most informative biomechanical predictors of tibial stress-fracture risk based on 500 linear mixed-effects (LME) models. Separately, for **(A, B)** men and **(C, D)** women, we computed the selection frequency of each biomechanical parameter in the subset of model predictors in the 500 “best” LME models (see **Figure 2**). **(A, C)** show the results when we considered all 30 kinematic and kinetic biomechanical parameters for model selection, while **(B, D)** show the results when we only considered the 14 kinematic parameters (first 14 parameters in **Table 2**). AD, ankle dorsiflexion; AP, ankle plantarflexion; ext, external; JRF, joint reaction force; rot, rotation.

The five-fold CV procedure repeated 100 times yielded a total of 500 LME models, which provided 100 independent SF-risk predictions for each participant to compute their ensemble-average SF risk (**Figure 1**, right panel; **Figure 2**). For men, when we considered all 30 modifiable gait parameters as potential predictors of tibial SF risk during model and parameter selection, the top three most frequently selected parameters included the knee stance flexion angle, hip external rotation angle, and ankle joint reaction force (JRF), which appeared in 38%–99% of the 500 models (**Figure 3A**). For women, the hip abduction and adduction moments as well as the ankle JRF were selected in 39%–76% of the models (**Figure 3C**).

In contrast, when we limited predictor selection to only the 14 kinematic parameters (rows 1–14 in **Table 2**), the hip external rotation, hip adduction, and ankle dorsiflexion angles were selected in only 14%–57% of the 500 models for men (**Figure 3B**), while the ankle plantarflexion, ankle dorsiflexion, and hip flexion angles were selected in 31%–79% of the models for women (**Figure 3D**). In summary, for both men and women, we found that the ankle JRF was the most informative predictor of tibial SF risk. However, when considering only kinematic parameters, we found that the ankle dorsiflexion angle was the most informative parameter for men and the second most informative for women, which had the hip flexion angle as their most informative kinematic parameter, while the hip external rotation angle was the second most informative parameter for men.

### 3.2 Performance of the personalized LME risk prediction models

**Figure 4:**
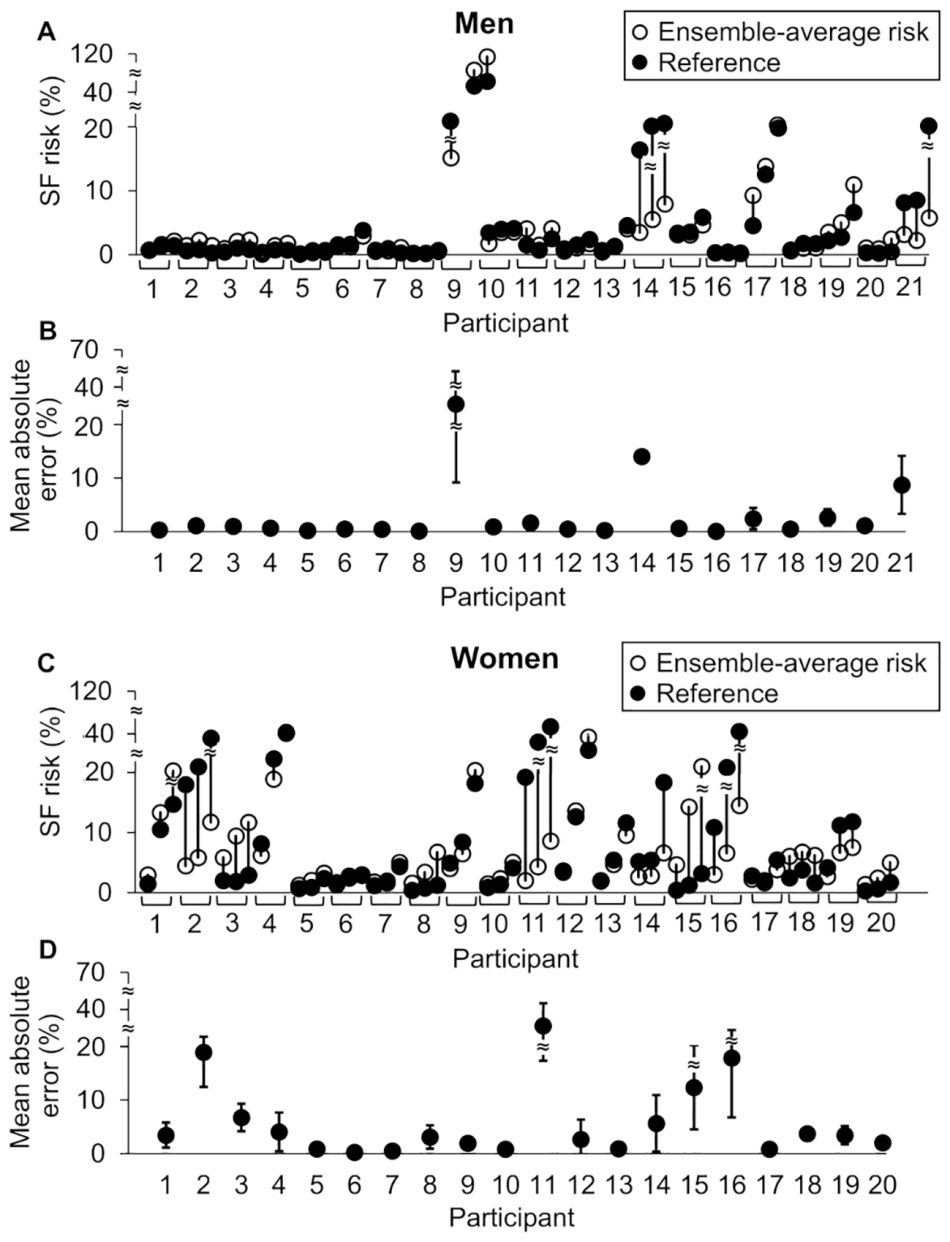
Comparison of stress-fracture (SF) risk between the reference values and the linear mixed-effects model ensemble-average predictions based on kinematic and kinetic parameters. Separately, for each load-carriage condition (i.e., 0.0, 11.3, or 22.7 kg) and for **(A)** each man and **(C)** each woman, we compared the personalized ensemble-average predictions based on 100 models per participant that considered all biomechanical parameters as potential predictors (open circles) and the reference (solid circles) based on the validated probabilistic model of SF risk. Panels **(B)** for men and **(D)** for women show the mean absolute error between the ensemble-average model results and the reference, averaged over the three load-carriage conditions per participant.

**Figure 5:**
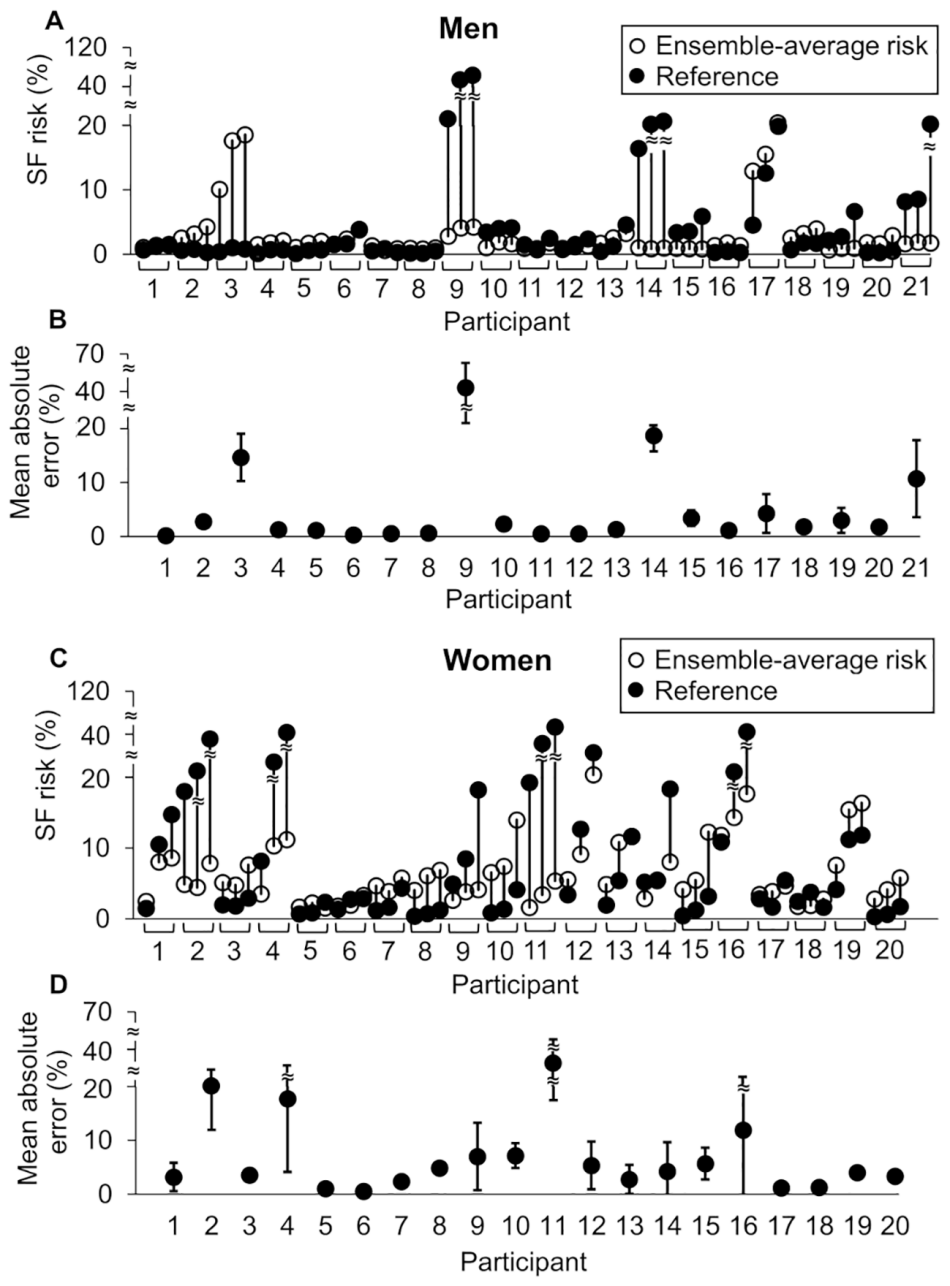
Comparison of stress-fracture (SF) risk between the reference values and the linear mixed-effects model ensemble-average predictions based solely on kinematic parameters. Separately, for each load-carriage condition (i.e., 0.0, 11.3, or 22.7 kg) and for **(A)** each man and **(C)** each woman, we compared the personalized ensemble-average predictions based on 100 models per participant that only considered the kinematic parameters as potential predictors (open circles) and the reference (solid circles) based on the validated probabilistic model of SF risk. Panels **(B)** for men and **(D)** for women show the mean absolute error between the ensemble results and the reference, averaged over the three load-carriage conditions per participant.

We assessed the performance of the LME models based on kinematic and kinetic parameters by comparing the ensemble-average SF-risk predictions with the corresponding reference SF-risk values. **Figures 4A, C** show the ensemble-average predicted SF risk for each of the 21 men and 20 women, for each of the three load-carriage conditions (0.0, 11.3, or 22.7 kg; open circles), along with their corresponding reference values (solid circles). **Figures 4B, D** show the associated MAE for the average discrepancy between the predicted and reference values over the three load conditions per participant. For both men and women, the ensemble models predicted the low range of reference SF risk (0% to 10%) reasonably well, with MAE values of less than ~3% for men and ~7% for women (except for Participant #15 whose MAE was 12%). In contrast, for reference SF risks over 10%, the predictions demonstrated participant-to-participant variability, with individual MAE values ranging from less than 1% up to 31%. As an example, for female Participants #4 and #12, who had two reference values exceeding 10%, the models predicted SF risk reasonably well with MAEs <4%. However, the models underpredicted the risk for Participants #2 and #11, resulting in MAEs as large as 31% (**Figures 4C, D**). For both the men and women, the ensemble models that only considered the 14 kinematic parameters performed slightly worse than those that considered all 30 parameters (**Figure 5**). For example, the models predicted the low range of reference SF risk (0% to 10%) with MAE values of less than ~15% for men and ~7% for women. For reference risks above 10%, we observed MAE values as large as 43% (**Figures 5B, D**). In summary, for both men and women, the ensemble-average models predicted participants at low risk better than those at high risk. Limiting the models to kinematic parameters only reduced prediction accuracy, which was more pronounced in men.

### 3.3 Performance of the binary LR classification models

**Figure 6:**
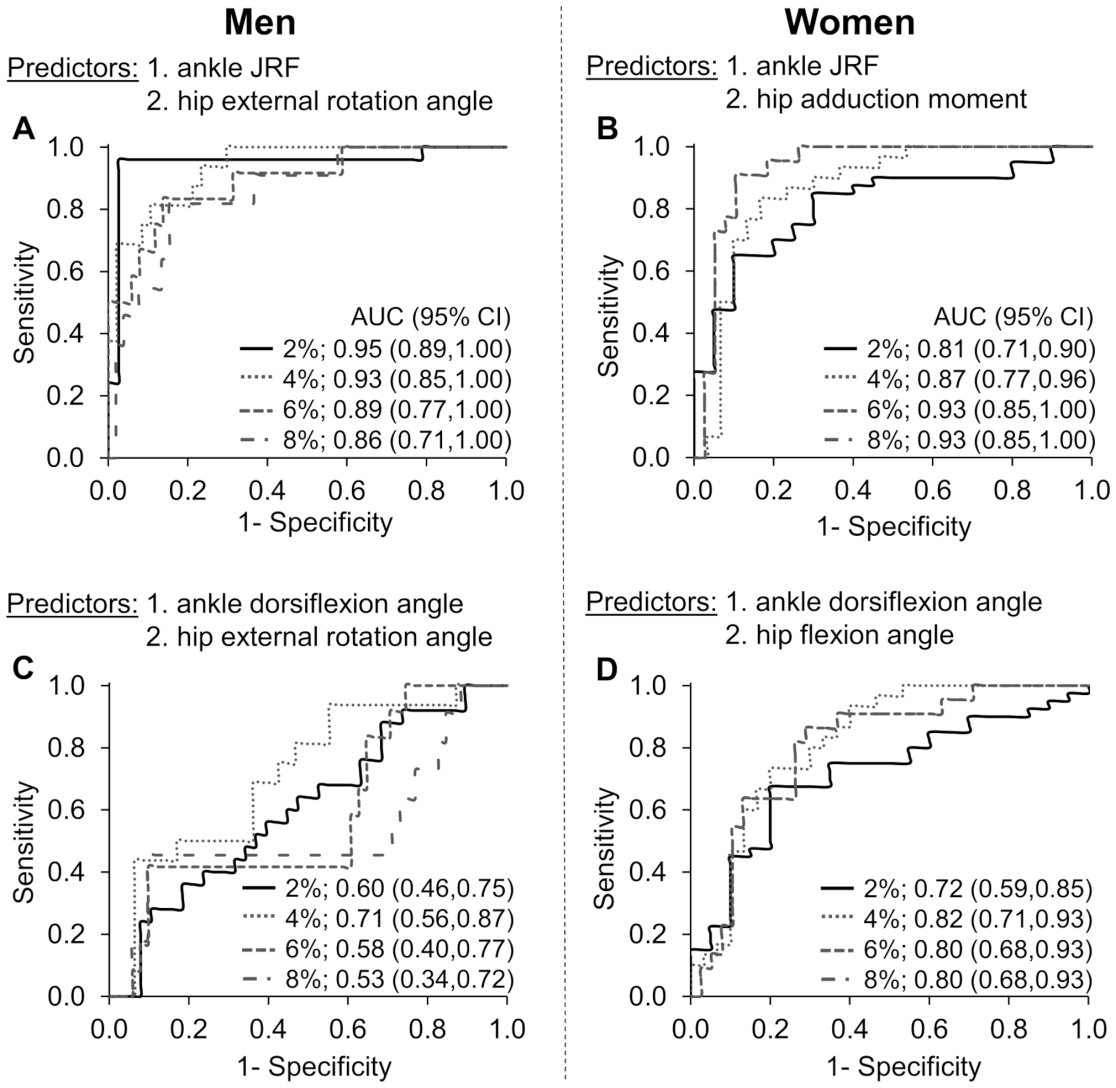
Assessment of the binary logistic regression (LR) models developed based on a five-fold cross-validation (CV) procedure repeated 100 times for various cutoffs of tibial stress-fracture (SF) risk to determine whether a participant had a low risk (negative) or a high risk (positive). The panels show the receiver operating characteristic area under the curve (AUC) for **(A, C)** men and **(B, D)** women for LR models based on the top two most informative biomechanical parameters selected through the CV procedure in **Figure 2**. For women (**B**, **D**), the curves for the 6% and 8% SF risk cutoffs overlapped with each other. **(A, B)** show the results when we considered all 30 biomechanical parameters for model selection, while **(C, D)** show the results when we only considered the 14 kinematic parameters. CI, confidence interval.

To discriminate participants at low risk from those at high risk for tibial SF injury, we separately developed and evaluated binary LR models for men and women using a five-fold CV procedure repeated 100 times. As predictors, we only used the top two most informative parameters obtained from the nested CV procedure of the LME models, out of all 30 biomechanical gait parameters or out of the 14 kinematic parameters. To assess the performance of the LR models, we computed the AUC for discriminatory SF-risk cutoffs of 2%, 4%, 6%, and 8%. For men, the combination of ankle JRF and hip external rotation angle yielded very accurate results for the probability of high SF risk, with AUCs ranging from 0.86 (CI: 0.71,1.00) to 0.95 (0.89,1.00). The AUCs decreased with increasing cutoff values (**Figure 6A**). When we used ankle dorsiflexion and hip external rotation angles as predictors in **Figure 6C**, we obtained considerably lower accuracy, with AUCs ranging from 0.53 (0.34,0.72) to 0.71 (0.56,0.87). Similarly, for women, the combination of ankle JRF and hip adduction moment yielded very accurate results, with AUCs ranging from 0.81 (0.71,0.90) to 0.93 (0.85,1.00). In this case, the AUCs increased with increasing cutoff values (**Figure 6B**). This trend is the opposite of that in men because in women we overpredicted risk for some of the low range reference SF values (e.g., Participants #3 and #15 in **Figure 4C**), leading to lower specificity and AUC for smaller cutoff values. Limiting the LR models to kinematic parameters (ankle dorsiflexion and hip flexion angles) yielded smaller AUCs, ranging from 0.72 (0.59,0.85) to 0.82 (0.71,0.93) (**Figure 6D**). Except for LR models for men based solely on kinematic parameters, modifiable gait parameters accurately discriminated a participant’s risk for tibial SF injury after a simulated 10-week BCT. In addition, the discrimination appeared to be largely insensitive to the selection of cutoff values between 4% and 8%.

## 4 Discussion

In this exploratory study, we developed statistical, data-driven models to predict an individual’s SF risk of the tibia and to classify the risk as low or high following a simulated 10-week military BCT regimen. Leveraging previously collected experimental laboratory data and reference SF risk values generated through a complex framework of personalized musculoskeletal, finite-element, and probabilistic computational models for 21 men and 20 women running on an instrumented treadmill while carrying an external load (Unnikrishnan et al., 2021; Rubio et al., 2023; Tong et al., 2023), we developed personalized LME models for men and women to identify sex-specific modifiable biomechanical gait parameters predictive of SF risk. Our results demonstrate that these simplified LME models can reasonably and more expeditiously approximate reference SF risk and adequately identify at-risk individuals, providing a computationally efficient alternative to the original more elaborate framework.

For the 21 men, the ensemble-average predictions on data not used for training the LME models yielded reasonable agreement with the reference SF risk, with MAEs ranging from less than 1% up to 31%, for an overall average of 3% (**Figures 4A, B**). For SF risk below the 10% cutoff, which encompassed 86% (54 out of 63) of the data points for men (**Supplementary Table S1**), the average of the absolute differences between the predicted and reference values was only 1% for the 54 data points. We observed even smaller discrepancies for lower cutoffs. However, the MAE above the 10% cutoff was considerably larger, with an average absolute difference of 17% for the nine data points above the cutoff, partially caused by Participant #9, who had absolute differences as large as 51%.

For the 20 women based on all 30 parameters, we observed considerably higher SF risk and a larger spread in both the predicted and reference data than those in men, with an overall average MAE of 6% (**Figures 4C, D**). As in men, the discrepancy between the predicted and the reference risk tended to increase with increasing cutoff values, as the number of data points above the cutoff decreased (**Supplementary Table S1**). For cutoffs up to 10%, which encompassed 67% (40 out of 60) of the data points for women, we observed an average absolute difference between the predicted and reference values of 3%. In contrast, for values larger than 10%, the average absolute difference was 13%, with the model underpredicting risk for Participants #2, #11, #16, and #19. In summary, for both men and women, the LME models based on combinations of subsets of all 30 parameters accurately predicted tibial SF risk for reference risk levels below 10%, which included the majority (~70%) of the participants. For context, theoretically, if the SF-risk cutoffs accurately characterized injury incidence, in our small dataset the 10% and 20% cutoffs for men would correspond to incidence rates of 19% and 14%, respectively. In women, these cutoffs would correspond to incidence rates of 50% and 25%, respectively.

Limiting the model predictors to the 14 kinematic parameters in **Table 2** increased predictive error for both men and women, however, the increase was larger in men than in women. For example, across all the men, the average MAE increased to 6% (from 3%, when models included both kinematic and kinetic parameters), and across all the women it increased to 7% (from 6%). This observation is not unexpected because kinetic parameters have been linked to SF risk of the tibia (Milner et al., 2006; Unnikrishnan et al., 2021; Milner et al., 2023; Rubio et al., 2023; Tong et al., 2023) and their absence as model predictors decreased predictive power, as indicated by the larger average MAEs.

Importantly, the development of these LME models also allowed us to identify the most informative (i.e., predictive) modifiable gait parameters (**Figures 3A, C**). Based on both kinematic and kinetic parameters, the nested five-fold CV procedure consistently identified three model parameters each for men and women out of the 500 (5 × 100) models. For men, the most frequently selected parameters included knee stance flexion angle (selected 38% of the time), hip external rotation angle (45%), and ankle JRF (99%), and for women, they included hip abduction moment (39%), hip adduction moment (64%), and ankle JRF (76%). None of the remaining 27 parameters appeared in more than 25% of the 500 models, providing confidence that the proposed approach had the ability to distinguish predictive signals from noise. In the absence of kinetic parameters, the procedure identified hip external rotation angle (14%), hip adduction angle (14%), and ankle dorsiflexion angle (57%) as the most predictive kinematic parameters for men (**Figure 3B**). However, only the ankle dorsiflexion angle appeared in more than 50% of the models, indicating the absence of parameters with strong predictive power. In contrast, for women the ankle plantarflexion angle (31%), ankle dorsiflexion angle (71%), and hip flexion angle (79%) stood out as the strongest predictive signals for tibial SF risk (**Figure 3D**).

Using the top two most informative parameters, we developed binary LR models to discriminate low- from high-risk individuals, for different high-risk cutoff values (**Figure 6**). Except for the models for men based solely on kinematic parameters, all the other models yielded adequate discrimination, with AUCs ranging from 0.72 to 0.95 and none of the 95% CIs crossing 0.50. Models whose CIs cross 0.50 do not provide adequate discrimination. For men and women, models that considered all parameters for all cutoff values yielded AUCs of at least 0.81 (**Figures 6A, B**). The LR models for men limited to kinematic parameters achieved AUCs ranging from 0.53 to 0.71, with only the model for the 4% high-risk cutoff yielding 95% CIs that did not cross 0.50 (**Figure 6C**). This poor performance is because the two most informative kinematic parameters used in the models (hip external rotation and ankle dorsiflexion angles; **Figure 3B**) were not strong predictors, appearing in only 14% and 57%, respectively, of the 500 LME models and implying a weak association with SF risk. These results suggest that kinematic parameters alone may be sufficient to identify women (but not men) at high risk for SF, whereas kinetic parameters provide additional discriminatory power for both men and women.

Some of the most informative kinetic and kinematic parameters identified in our analysis have been previously linked to the development of tibial SF or bone stress injuries. For example, ankle JRF, a predictor in over 75% of the 500 LME models for both men and women (**Figure 3**), represents a major component of the load to the tibia (Brockett and Chapman, 2016). Indeed, an elevated ankle JRF has been associated with the development of tibial SF injury (Milner et al., 2006, Milner et al., 2023) and was reported to be higher in female runners with a history of tibial SF injury than in healthy controls (Meardon et al., 2015). Hip adduction moment, a predictor in 64% of the women’s LME models, has been theorized to cause abnormal tibial loading (Powers, 2010) and was reported to be higher in female runners with a history of tibial SF injury than in age- and mileage-matched controls (Pohl et al., 2008). Among the joint angles, ankle dorsiflexion, a predictor in over 57% of the models for both men and women, has been reported to be critical for tibial loading. Specifically, in a study of healthy adults walking and jogging, those with small ankle dorsiflexion angles displayed alterations in hip and pelvis kinematics, including reduced hip extension and pelvic rotation, which correlate with lower-extremity injury (Rao et al., 2024).

This work offers new insights and potentially actionable information for future studies. In our analysis, we identified a positive association between tibial SF risk and hip external rotation angle in men and between tibial SF risk and hip flexion angle in women. Although we found no previous reports associating either parameter with tibial loading or SF, these results suggest that future experimental studies could investigate these hypotheses. In theory, we could assess the gait parameters of military recruits before the start of BCT and use an ensemble-average of the SF risk predictions from all 500 LME models to estimate their tibial SF risk. However, it is not practical to assess thousands of recruits, and the approach is not suitable for field use because the kinetic parameters require the development of musculoskeletal models. We could circumvent this by considering only joint angles as predictors, which can be measured in the field with wearable IMUs (Seel et al., 2014). Park and Yoon reported that the accuracy of IMUs is comparable to that of a laboratory three-dimensional motion-capture system (Kobsar et al., 2020; Park and Yoon, 2021; Hafer et al., 2023), eliminating the need for time-consuming, laboratory-based measurements and kinetic parameter value estimation. Limiting predictors to joint angles would reduce model accuracy, but in exchange, it would provide faster, real-time SF risk assessments in the field. If, however, the goal is to screen high-risk recruits, at least for women, measuring only ankle and hip angles with IMUs and using LR models would adequately identify high-risk participants (**Figure 6**). We recognize that these suggested potential applications are contingent upon independent validation of our findings and a demonstrated association between these risk estimates and actual incidence of SF injuries.

This study has limitations. First, even though the reference SF risk previously estimated by the probabilistic model does not correspond to a clinical SF incident, we assumed that it is sufficiently accurate and reflects the participants’ true underlying risk. We believe that this is a reasonable assumption because the inputs to this model are based on personalized measurements and predictions. In addition, the probabilistic model itself accounts for bone repair and adaptation based on experimental data and considers bone fatigue using a physics-based fatigue-damage framework (Taylor et al., 2004; Edwards et al., 2010). Importantly, the model results are supported by observations from large epidemiological studies involving hundreds to thousands of participants in a 10-week BCT regimen. For example, supported by multiple sources of evidence (Knapik et al., 2012; Molloy et al., 2020; Kardouni et al., 2021), the model estimated the probability of tibial SF risk in women to be approximately twice of that in men, with an average of 9.7% [standard deviation (SD)=12.6%] for women and 5.6% (SD = 11.3%) for men. These estimates are within or close to the medically diagnosed SF incidence ranges of 1.1% to 18.0% for women and of 0.8% to 5.1% for men (Knapik et al., 2012) and consistent with observations that both the incidence rate (2.0%) and adjusted risk ratio (6.1) of lower-leg SF in women are considerably higher than the corresponding values in men (0.7% and 3.6, respectively) (Kucera et al., 2016). Second, our predictions of informative modifiable gait parameters and model assessments are only valid for the current dataset. While we repeated the five-fold CV procedure 100 times and assessed model performance with different test subsets not used for training the models, without independent evaluation of model performance using new data we cannot establish generalizability. Third, the small sample size of the datasets for men (N = 21) and women (N = 20) restricted our LME models to a maximum of five predictors to avoid model overfitting. Fourth, because we used the full dataset to identify top predictors via the fully nested LME framework and then used those same predictors for our LR models, the resulting AUCs may be optimistically biased. Fifth, because we utilized observation-level data to classify the reference SF-risk values as “high” or “low” for the LR models, the resulting CIs for the AUCs may be narrower than expected due to potential correlations of the data points within participants. Finally, because using SF risk cutoffs over 8% resulted in severely imbalanced datasets below and above the cutoff (**Supplementary Table S1**), we had to limit the analysis of the LR models to this cutoff. Future studies with larger datasets should allow for more robust model training and improved generalizability.

## 5 Conclusion

We developed a capability to identify modifiable gait parameters predictive of SF risk, estimate individual tibial SF risk, and flag susceptible individuals. In the future, upon validation with new data, this work could help target recruits who may benefit from individualized training regimens, ultimately facilitating proactive interventions to reduce injury risk.

## Author‘s note

We independently assessed the results presented herein for reproducibility. The opinions and assertions contained herein are the private views of the authors and are not to be construed as official or as reflecting the views of the Defense Health Agency, the U.S. Department of Defense, the U.S. Government, or The Henry M. Jackson Foundation for the Advancement of Military Medicine, Inc. This paper has been approved for public release with unlimited distribution.

## Data Availability

The original contributions presented in the study are included in the article/**Supplementary Material**. Further inquiries can be directed to the corresponding author.
