# Supplementary material for: Identification of modifiable gait parameters predictive of simulated tibial stress-fracture risk in men and women
Source: Front Physiol. 2026 Aug 5;17:1905928. doi: 10.3389/fphys.2026.1905928 (PMC13485774; doi:10.3389/fphys.2026.1905928)
Supplement: Supplementary file 1 [file DataSheet1.docx]

**Identification of Modifiable Gait Parameters Predictive of Simulated Tibial Stress-Fracture Risk in Men and Women**

**Sridevi Nagaraja^1,2^, Jose E. Rubio^1,2^, Manivannan Subramaniyan^1,2^, Junfei Tong^1,2^, Michael Baggaley^3,4^, W. Brent Edwards^3,4^, and Jaques Reifman^1,*^**

^1^Department of Defense Biotechnology High Performance Computing Software Applications Institute, Defense Health Agency Research & Development, Medical Research and Development Command, Fort Detrick, MD, USA

^2^The Henry M. Jackson Foundation for the Advancement of Military Medicine, Inc., Bethesda, MD, USA

^3^Human Performance Laboratory, Faculty of Kinesiology, University of Calgary, Alberta, Canada

^4^The McCaig Institute for Bone and Joint Health, Cumming School of Medicine, University of Calgary, Alberta, Canada

*** Correspondence:**
Jaques Reifman

jaques.reifman.civ@health.mil

Supplementary Material

# 1 Supplementary Table

| TABLE S1 Data Splits for the Men and Women Datasets for Different Stress-Fracture (SF) Risk Cutoff Values | | | | |
| --- | --- | --- | --- | --- |
| **SF risk**  **cutoff (%)** | **Men (63 data points)** | | **Women (60 data points)** | |
|  | **Below**  **(low risk)** | **Above**  **(high risk)** | **Below**  **(low risk)** | **Above**  **(high risk)** |
| 2 | 38 | 25 | 20 | 40 |
| 4 | 47 | 16 | 30 | 30 |
| 6 | 51 | 12 | 38 | 22 |
| 8 | 52 | 11 | 38 | 22 |
| 10 | 54 | 9 | 40 | 20 |
| 12 | 54 | 9 | 45 | 15 |
| 14 | 55 | 8 | 46 | 14 |
| 16 | 55 | 8 | 47 | 13 |
| 18 | 56 | 7 | 47 | 13 |
| 20 | 57 | 6 | 51 | 9 |
